# Simian-Human Immunodeficiency Virus SHIV.C.CH505 Persistence in ART-Suppressed Infant Macaques Is Characterized by Elevated SHIV RNA in the Gut and a High Abundance of Intact SHIV DNA in Naive CD4^+^ T Cells

**DOI:** 10.1128/JVI.01669-20

**Published:** 2020-12-22

**Authors:** Veronica Obregon-Perko, Katherine M. Bricker, Gloria Mensah, Ferzan Uddin, Mithra R. Kumar, Emily J. Fray, Robert F. Siliciano, Nils Schoof, Anna Horner, Maud Mavigner, Shan Liang, Thomas Vanderford, Julian Sass, Cliburn Chan, Stella J. Berendam, Katharine J. Bar, George M. Shaw, Guido Silvestri, Genevieve G. Fouda, Sallie R. Permar, Ann Chahroudi

**Affiliations:** a Department of Pediatrics, Emory University School of Medicine, Atlanta, Georgia, USA; b Department of Medicine, Johns Hopkins University School of Medicine, Baltimore, Maryland, USA; c Howard Hughes Medical Institute, Baltimore, Maryland, USA; d Yerkes National Primate Research Center, Emory University, Atlanta, Georgia, USA; e Department of Biostatistics and Bioinformatics, Duke University Medical Center, Durham, North Carolina, USA; f Duke Human Vaccine Institute, Duke University Medical Center, Durham, North Carolina, USA; g Perelman School of Medicine, University of Pennsylvania, Philadelphia, Pennsylvania, USA; h Center for Childhood Infections and Vaccines of Children’s Healthcare of Atlanta and Emory University, Atlanta, Georgia, USA; Icahn School of Medicine at Mount Sinai

**Keywords:** pediatric, cure, human immunodeficiency virus, nonhuman primates, reservoir

## Abstract

Mother-to-child transmission of human immunodeficiency virus type 1 (HIV-1) continues to cause new pediatric cases of infection through breastfeeding, a setting where it is not always possible to initiate early antiretroviral therapy (ART). Without novel interventions that do not rely on daily ART, HIV-1-infected children face lifelong medications to control infection. A detailed analysis of virus persistence following breast milk transmission of HIV-1 and ART has not been performed. Here, we used infant rhesus macaques orally infected with simian/human immunodeficiency virus (SHIV) (SHIV.C.CH505) to identify cellular and anatomical sites of virus persistence under ART. Viral DNA was detected at similar levels in blood and tissue CD4^+^ T cells after a year on ART, with virus in blood and lymphoid organs confirmed to be replication competent. Viral RNA/DNA ratios were elevated in rectal CD4^+^ T cells compared to those of other sites (*P ≤ *0.0001), suggesting that the gastrointestinal tract is an active site of virus transcription during ART-mediated suppression of viremia. SHIV.C.CH505 DNA was detected in multiple CD4^+^ T cell subsets, including cells with a naive phenotype (CD45RA^+^ CCR7^+^ CD95^–^). While the frequency of naive cells harboring intact provirus was lower than in memory cells, the high abundance of naive cells in the infant CD4^+^ T cell pool made them a substantial source of persistent viral DNA (approximately 50% of the total CD4^+^ T cell reservoir), with an estimated 1:2 ratio of intact provirus to total viral DNA. This viral reservoir profile broadens our understanding of virus persistence in a relevant infant macaque model and provides insight into targets for cure-directed approaches in the pediatric population.

**IMPORTANCE** Uncovering the sanctuaries of the long-lived HIV-1 reservoir is crucial to develop cure strategies. Pediatric immunity is distinct from that of adults, which may alter where the reservoir is established in infancy. Thus, it is important to utilize pediatric models to inform cure-directed approaches for HIV-1-infected children. We used an infant rhesus macaque model of HIV-1 infection via breastfeeding to identify key sites of viral persistence under antiretroviral therapy (ART). The gastrointestinal tract was found to be a site for low-level viral transcription during ART. We also show that naive CD4^+^ T cells harbored intact provirus and were a major contributor to blood and lymphoid reservoir size. This is particularly striking, as memory CD4^+^ T cells are generally regarded as the main source of latent HIV/simian immunodeficiency virus (SIV) infection of adult humans and rhesus macaques. Our findings highlight unique features of reservoir composition in pediatric infection that should be considered for eradication efforts.

## Abstract (precis)

Uncovering the sanctuaries of the long-lived HIV-1 reservoir is crucial to develop cure strategies. Pediatric immunity is distinct from that of adults, which may alter where the reservoir is established in infancy. Thus, it is important to utilize pediatric models to inform cure-directed approaches for HIV-1-infected children. We used an infant rhesus macaque model of HIV-1 infection via breastfeeding to identify key sites of viral persistence under antiretroviral therapy (ART). The gastrointestinal tract was found to be a site for low-level viral transcription during ART. We also show that naive CD4^+^ T cells harbored intact provirus and were a major contributor to blood and lymphoid reservoir size. This is particularly striking, as memory CD4^+^ T cells are generally regarded as the main source of latent HIV/simian immunodeficiency virus (SIV) infection of adult humans and rhesus macaques. Our findings highlight unique features of reservoir composition in pediatric infection that should be considered for eradication efforts.

## INTRODUCTION

At the end of 2019, an estimated 1.8 million children were living with human immunodeficiency virus type 1 (HIV-1) infection worldwide, with 150,000 new cases in that year alone (UNAIDS Global HIV & AIDS statistics at https://www.unaids.org/en/resources/fact-sheet, accessed 19 July 2020). Current antiretroviral therapy (ART)-based strategies to prevent mother-to-child transmission (MTCT) can be limited by implementation challenges (access to ART, adherence to ART, and late presentation to prenatal care) (1, 2). Such obstacles contribute to new or untreated maternal HIV-1 infections in the peripartum or postpartum period, posing a risk for transmission through breastfeeding, a route that now accounts for the majority of new pediatric infections (3). Infants who acquire HIV-1 infection are committed to lifelong therapy. While early and very early initiations of ART in perinatally infected children have been associated with smaller reservoirs following ART interruption (4), this strategy is not always feasible to implement in the setting of breast milk transmission, which can be accompanied by delayed diagnosis. Thus, there is a critical need to develop novel interventions that can complement or serve as alternatives to ART-based measures to achieve remission or cure in HIV-1-infected children.

The major obstacle to clearing HIV infection is the early establishment of the long-lived viral reservoir, which persists in infected cells even in the absence of active virion production (5, 6). The reservoir consists of latently infected cells containing integrated HIV-1 provirus that are not cleared by ART and are capable of reactivation if therapy is discontinued. As such, uncovering the cellular and anatomic sources of virus responsible for rebound viremia remains paramount to cure research. Studies of cellular reservoirs have found HIV-1 in all memory subsets of CD4^+^ T cells, but central, transitional, and effector memory cells are most frequently infected and are regarded as key cell populations involved in maintaining viral persistence (7–12). Naive CD4^+^ T cells have also been highlighted as a potential reservoir, although it remains less clear how and when they become infected and whether they contribute to viral rebound upon ART interruption (13–15). Characterization of anatomical reservoirs in humans and animal models has demonstrated enrichment of virus within lymphoid organs and the gut mucosa (16–20). High viral burden at specific tissue sites may be partially explained by differences in CD4^+^ T cell composition, as some tissues have a higher abundance of cell subsets frequently infected by HIV-1 (21, 22). In addition, suboptimal ART penetration may foster anatomical sanctuaries for viral persistence (23, 24).

It is important to note that comprehensive assessments of HIV-1 reservoir composition have focused largely on adults, with similar studies of infants and children generally lacking. The distinct features of pediatric immunity and infection may impact where the virus persists or how latency is maintained early in life differently than when infection is acquired in adulthood. Notably, the immune system in the early years of life is tolerogenic but faces bursts of immune activation from vaccinations and microbial exposures throughout childhood (25–27). In addition, CD4^+^ T cell turnover rates and the proportion of naive cells are higher in children than in adults (27). The tempo of disease in HIV-1-infected children is also altered, as they sustain high levels of viremia after the peak, with approximately 50% of untreated children progressing to death within 2 years of infection (28). Transmission through the oral route is a highly relevant, yet understudied, mode of infection in infants through breastfeeding (29). For these age-related distinctions, child-specific investigations of HIV-1 persistence are critically needed.

The simian immunodeficiency virus (SIV) rhesus macaque model has been a mainstay in HIV-1 research for its many similarities to HIV-1 transmission and pathogenesis in humans (30–33). Our laboratory recently published the development of an infant rhesus macaque model of oral SIV infection and long-term ART to investigate child-specific features of virus persistence (34). Here, we sought to confirm and expand our previous findings with a model of simian/human immunodeficiency virus (SHIV) infection, using SHIV.C.CH505.375H.dCT, a chimeric virus expressing a clade C HIV Env engineered for enhanced replication and pathogenesis in rhesus macaques that is suitable for testing HIV envelope-targeting cure strategies (35). This model was used to interrogate viral reservoirs in blood and tissues longitudinally as well as upon sacrifice following long-term suppression of viremia with ART. Our findings add to the currently limited knowledge of reservoir composition in the setting of oral virus transmission.

## RESULTS

### Oral SHIV.C.CH505 infection of infant macaques.

**FIG 1:**
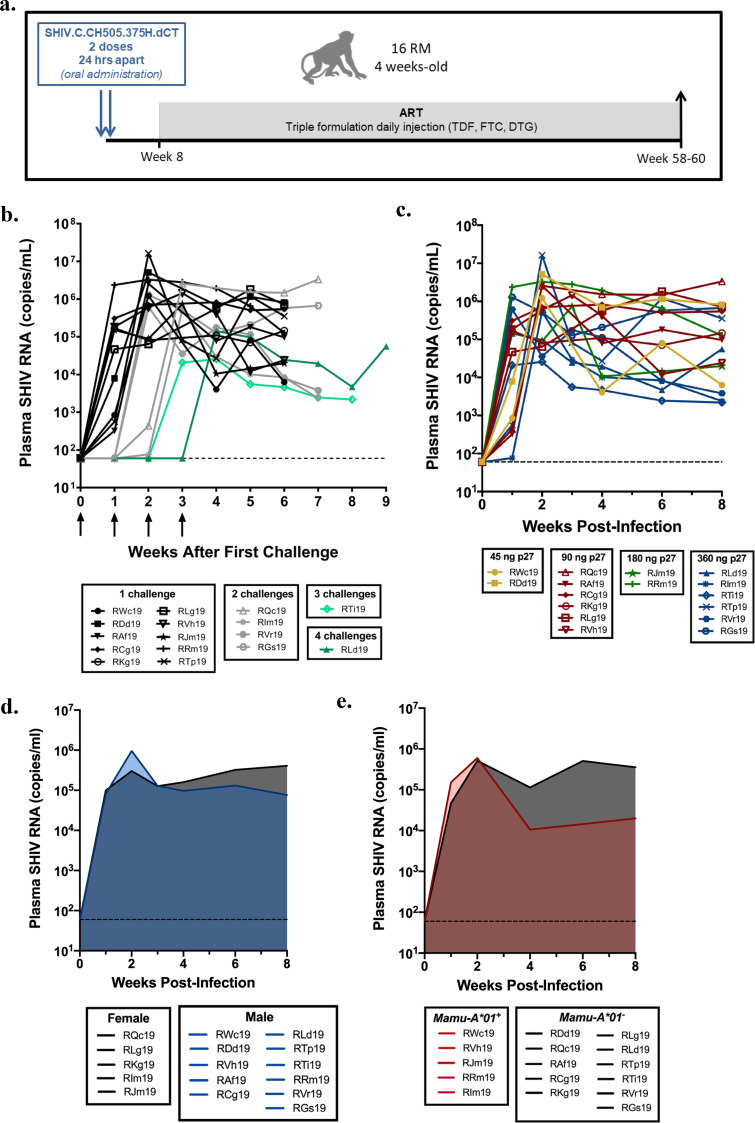
Infant rhesus macaque model of oral SHIV.C.CH505 infection. (a) Study schematic showing timing and duration of ART. (b to e) Viral replication kinetics prior to ART assessed by number of challenges (b), challenge dose (c), sex (d), and Mamu-A*01 status (e). In panel b, arrows indicate challenge. Plasma viral loads were determined by real-time RT-PCR (*n *= 16). In panels b and c, each curve represents one animal. Shaded regions in panels d and e represent the median.

We first set out to identify an oral inoculation dose that would yield a high infection rate to confine challenges to early infancy, with the objective of achieving infection during a time frame that models the early breastfeeding period. Infant rhesus macaques were inoculated by the oral route with SHIV.C.CH505 starting at 4 weeks of life (Fig. 1a). Animals were challenged on a rolling basis throughout the macaque birthing season as they reached the target age for enrollment into the study. We began with a challenge dose of 45 ng p27, roughly 5-fold higher than the dose previously shown to infect adult rhesus macaques by a single intravenous (i.v.) challenge with the same stock of virus (36). Two of the first four challenged infant macaques became viremic by 1 week postchallenge (50% infection rate) (Table 1). To increase the infection rate, the dose was then escalated to 90 ng p27 to challenge the next six animals and to rechallenge the two aviremic animals. Inoculation doses were increased for subsequent challenges of new animals until all 16 animals were infected. While most infant macaques were infected at lower doses, 360 ng p27 was ultimately needed to infect the two animals that were resistant to three or more prior challenges. This refraction to infection was mirrored by dampened peak and set point viral loads in these animals (RLd19, RTi19) (Fig. 1b). Across all macaques, peak viremia was usually seen at 2 to 3 weeks postinfection (wpi; the infection date was set to the challenge date that preceded first viremia time point), after which viral loads were sustained at near-peak levels or gradually declined. These replication kinetics are consistent with observations of untreated pediatric HIV-1 infection and contrast with the more rapid post-peak decline observed in HIV-1-infected adults (37, 38).

**TABLE 1:** Infection rate by challenge dose

Macaque	Detection of SHIV.C.CH505 in animals challenged with the indicated inocula (ng of p27)^*a*^				
	45/45, 2/4 macaques (50%)	90/90, 6/8 macaques (75%)	180/180, 2/5 macaques (40%)	360/360,^*b*^ 4/6 macaques (66%)	360/360,^*c*^ 2/2 macaques (100%)
RWc19	**+**				
RDd19	**+**				
RLd19	**–**	**–**	**–**	**+**	
RQc19	**–**	**+**			
RAf19		**+**			
RCg19		**+**			
RKg19		**+**			
RLg19		**+**			
RVh19		**+**			
RTi19		**–**	**–**	**+** (180/360)	
RJm19			**+**		
RRm19			**+**		
RIm19			**–**	**+** (180/360)	
RTp19				**+** (180/360)	
RVr19				**–**	**+**
RGs19				**–**	**+**

a +, detectable viral loads by 7 days p.i.; –, undetectable viral loads by 7 days p.i.

b Numbers in parentheses are modified doses.

c For animals rechallenged with 360 ng p27, the dose was not escalated.

There were no dose-dependent effects on viral replication kinetics or levels (*P = *0.38) (Fig. 1c), and males and females were found to have similar viral dynamics (*P = *0.39) (Fig. 1d). Expression of several major histocompatibility complex (MHC) class I alleles has previously been associated with slow disease progression and natural control of viral replication in HIV-1 and SIV infection (39, 40). Infant macaques were selected to be Mamu-B*08-/B*17- to exclude controller phenotypes. We included both Mamu-A*01^–^ and -A*01^+^ animals, as the latter allele has been valuable in evaluating epitope-specific T cell responses in preclinical macaque studies (41, 42). In our model, Mamu-A*01^+^ macaques were equally susceptible to infection but had set point viral loads approximately 1 log lower than those of Mamu-A*01^–^ animals (*P = *0.06) (Fig. 1e).

### Viral and immune dynamics on long-term ART.

**FIG 2:**
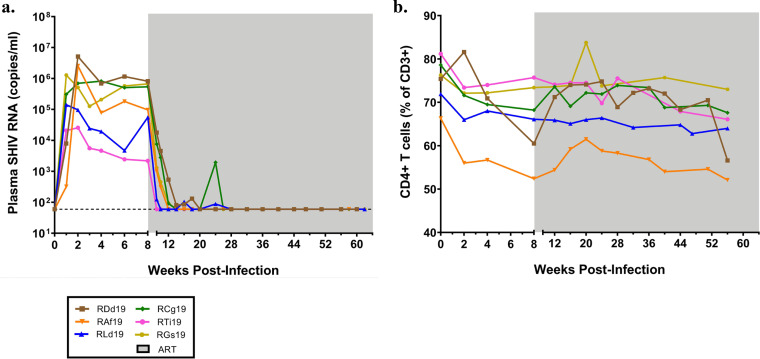
Viral loads and CD4^+^ T cell frequencies in macaques on long-term ART. (a) Plasma viral loads measured longitudinally by real-time RT-PCR. The dashed line indicates the LOD. (b) Peripheral CD4^+^ T cell frequency determined by flow cytometry staining of whole blood. Each curve represents one animal, and the shaded region indicates the weeks that the animals were on ART (*n *= 6).

All SHIV.C.CH505-infected rhesus macaques were initiated on a daily ART regimen consisting of tenofovir disoproxil fumarate (TDF), emtricitabine (FTC), and dolutegravir (DTG) given by subcutaneous injection starting at 8 wpi. Six animals, chosen to represent a range of peak and pre-ART viral loads and used henceforth for viral reservoir measurements, were maintained on ART for a minimum of 50 weeks prior to the endpoint of this study (Fig. 2a). ART was effective at reducing viral replication in plasma to levels below the limit of detection of our assay (60 copies/ml) within 1 to 12 weeks (Fig. 2a). A longer time to suppression of viremia was seen in macaques with higher viral loads at the time of ART initiation. ART-mediated suppression of viremia was durable, as there were no detectable viral blips for at least 8 months preceding the study endpoint.

Longitudinal tracking of CD4^+^ T cell frequencies in peripheral blood revealed an average decline of 8.9% (standard deviation [SD], ±4.9) from the day of infection to ART initiation at 8 wpi (Fig. 2b). Animals with greater declines also had the highest viral loads at ART initiation, but frequencies typically returned to near baseline during ART. One animal, RDd19, showed an atypical CD4^+^ T cell reduction toward the end of the study but also had non-SIV-related health complications at that time (idiopathic demyelinating disease), which may have affected lymphocyte composition.

### SHIV.C.CH505 DNA and RNA persistence across anatomical sites.

**FIG 3:**
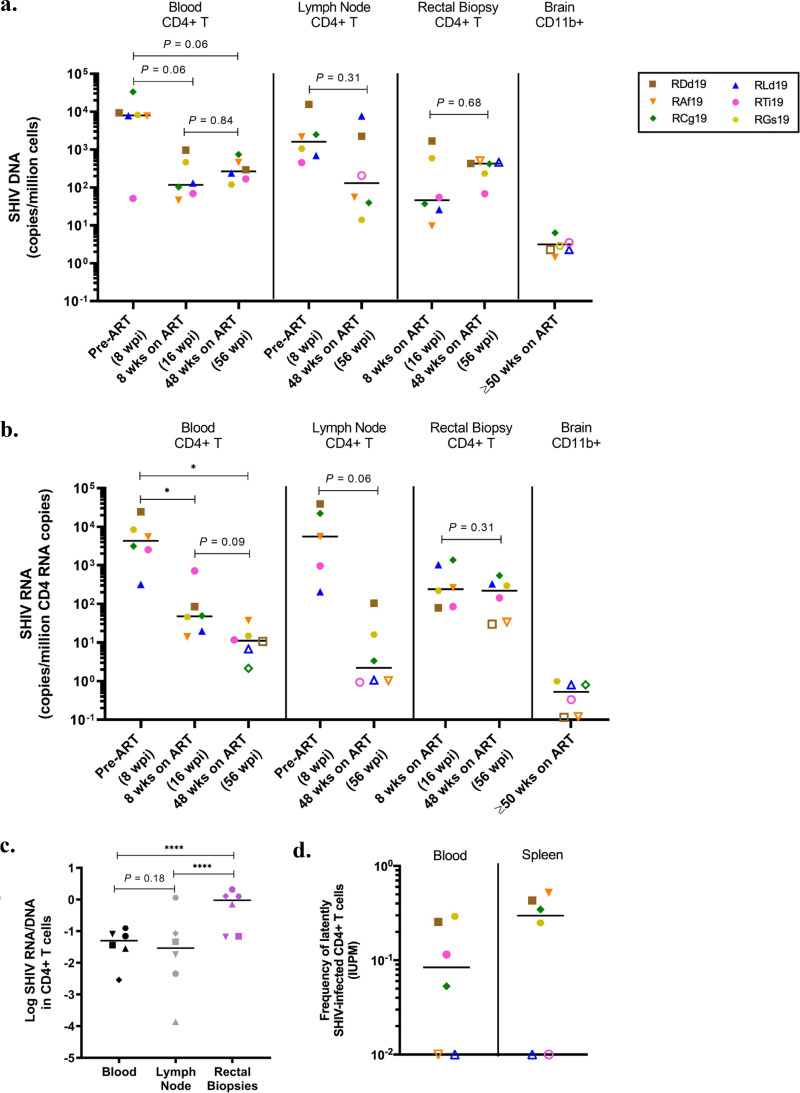
SHIV.C.CH505 persistence in blood and tissue CD4^+^ T cells. (a and b) Cell-associated SHIV DNA (a) and SHIV RNA (b) in enriched CD4^+^ T or CD11b^+^ cells at the indicated time points. Statistical significance was determined by the Wilcoxon signed-rank test. (c) Log-transformed RNA/DNA ratios in CD4^+^ T cells from the indicated sites after 48 weeks on ART. The Wilcoxon signed-rank test was used for statistical analysis. (d) Replication-competent virus levels in enriched blood and spleen CD4^+^ T cells after ≥50 weeks on ART as determined by QVOA. In all panels, each point represents one animal (*n *= 6), and the line is at the median. Open symbols indicate values below the LOD. *, *P < *0.05; ****, *P < *0.0001.

While previous studies have evaluated viral distribution shortly after oral SHIV/SIV challenge in infant macaques (43–46), less is known about the anatomical location of reservoirs during long-term ART and whether viral burdens are comparable between sites. To longitudinally track SHIV.C.CH505 levels, we quantified total cell-associated viral DNA in CD4^+^ T cells isolated from blood, lymph nodes, and rectal biopsy specimens (Fig. 3a). As expected, levels of SHIV.C.CH505 DNA were highest prior to ART initiation (8 wpi) in blood and lymph node CD4^+^ T cells. A trend for decay of viral DNA in the periphery was seen by 8 weeks on ART (16 wpi), after which levels of CD4^+^ T cells containing viral DNA remained stable through the next 40 weeks. Similarly, in the lymph nodes, SHIV.C.CH505 DNA levels were typically reduced by 1 to 2 logs after 48 weeks on ART compared to pre-ART values. Due to the small size of infant macaques, rectal biopsy specimens could not be safely collected until 16 wpi, 8 weeks after ART was initiated. Thus, we could not evaluate SHIV.C.CH505 DNA levels in CD4^+^ T cells from this compartment pre-ART but were able to observe stability from 8 to 48 weeks on ART, similar to findings for peripheral blood. To evaluate viral persistence in the central nervous system (CNS) at the study endpoint (≥50 weeks on ART), CD11b^+^ myeloid cells were isolated from the brain and viral DNA was quantified. SHIV.C.CH505 DNA was detected at very low levels (1 to 6 copies/million CD11b^+^ cells) in only two of six rhesus macaques (Fig. 3a).

We next quantified SHIV.C.CH505 RNA expression in CD4^+^ T cells and CD11b^+^ myeloid cells from the same four sites. SHIV.C.CH505 RNA could be quantified in CD11b^+^ cells isolated from the brain of only one animal (Fig. 3b). In contrast, with this model and virus, CD4^+^ T cells containing viral RNA were readily detected. SHIV.C.CH505 RNA levels in blood and lymph node CD4^+^ T cells were largely reduced between the pre-ART time point and 48 weeks on ART (*P = *0.03 and *P = *0.06, respectively) (Fig. 3b). In CD4^+^ T cells from rectal biopsy specimens, cell-associated viral RNA remained stable from 8 weeks to 48 weeks on ART (median of 505 to 228 SHIV.C.CH505 copies/million CD4 copies, respectively; *P = *0.31). Moreover, SHIV.C.CH505 RNA expression in rectal CD4^+^ T cells was approximately 1 log higher than in cells from the blood and lymph node after 48 weeks on ART, despite all compartments having similar SHIV.C.CH505 DNA levels at the same time point. Indeed, when comparing the median RNA/DNA ratios across these sites, relative SHIV.C.CH505 RNA expression was significantly higher in rectal CD4^+^ T cells (*P ≤ *0.0001) (Fig. 3c), highlighting this tissue as a site of active virus transcription in infant macaques during ART.

Measurements of total cell-associated viral DNA represent both defective and intact replication-competent provirus. We therefore asked if the SHIV.C.CH505 DNA in CD4^+^ T cells (shown in Fig. 3a) reflected the presence of replication-competent virus. CD4^+^ T cells were enriched from tissues harvested at necropsy (≥50 weeks on ART) and assessed by a quantitative viral outgrowth assay (QVOA). Low cell numbers from the gastrointestinal tract limited our analysis to blood and lymphoid compartments. Infectious virus was detected at frequencies ranging from 0.05 to 0.29 and 0.25 to 0.52 infectious units per million (IUPM) cells from blood and spleen, respectively (Fig. 3d). With a single round of anti-CD3/CD28 stimulation, infectious virus could not be cultured from blood and spleen in one animal (RLd19). In two animals, infectious virus was cultured from one but not both sites (RAf19 and RTi19).

### Contribution of CD4^+^ T cell subsets to the SHIV.C.CH505 reservoir.

**FIG 4:**
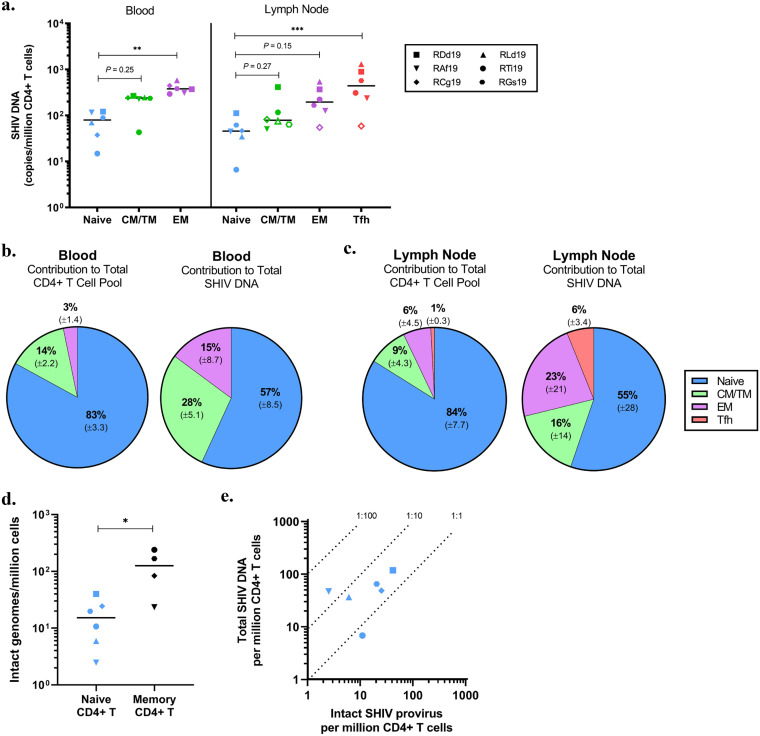
Contribution of CD4^+^ T cell subsets to the total SHIV.C.CH505 reservoir. (a) Cell-associated SHIV DNA in CD4^+^ T cells subsets sorted from PBMC and lymph nodes at ≥32 weeks on ART. Open symbols indicate values below the LOD (*n *= 6). The Friedman test with Dunn’s correction was used for statistical analysis. (b and c) Average contributions of CD4^+^ T cell subsets to the total CD4^+^ T cell pool and SHIV DNA persistence in blood (b) and lymph node (c) (*n *= 6). Percentages reflect means, with the SD indicated below. (d) Intact genomes, as determined by IPDA, in naive (*n *= 6) and bulk memory (*n *= 4) CD4^+^ T cells enriched from lymph nodes at ≥50 weeks on ART. The Mann-Whitney test was used to determine statistical significance. (e) Ratios of intact provirus to total SHIV DNA in naive CD4^+^ T cells. In all panels, each point represents one animal, with the line at the median. CM, central memory; TM, transitional memory; EM, effector memory; Tfh, T follicular helper. *, *P < *0.05; **, *P < *0.01; ***, *P < *0.001.

We recently demonstrated that naive CD4^+^ T cells are a major contributor to SIV DNA persistence and harbor replication-competent virus in SIV-infected ART-treated infant macaques (34). Thus, we aimed to corroborate and expand these findings in the SHIV.C.CH505 model. CD4^+^ T cells were isolated from blood and lymph nodes collected after ≥32 weeks on ART and then sorted into T cell subsets before we quantified SHIV.C.CH505 DNA levels. As in previous reports (7, 9), viral DNA was enriched in differentiated memory subsets, such as effector memory cells (CD45RA^−^ CCR7^−^ CD95^+^), and within T follicular helper (Tfh) cells (CXCR5^hi^ PD-1^hi^) in the lymph node compared to levels in naive CD4^+^ T cells (CD45RA^+^ CCR7^+^ CD95^−^) (Fig. 4a). However, levels of SHIV.C.CH505 DNA in naive CD4^+^ T cells were comparable to those in cells with a central/transitional memory phenotype (CD45RA^−^ CCR7^+^ CD95^+^). When considering both the relative frequency in the CD4^+^ T cell pool and the levels of viral DNA within each subset, naive CD4^+^ T cells are estimated to comprise over half of the total SHIV.C.CH505 DNA burden in peripheral CD4^+^ T cells (Fig. 4b). Similar findings were obtained in the lymph node, although there was greater variability of SHIV.C.CH505 DNA levels between animals (Fig. 4c).

To further interrogate the naive CD4^+^ T cell reservoir in infant macaques, we used the intact proviral DNA assay (IPDA), a PCR technique previously demonstrated to specifically quantify intact genomes (47, 48). Intact proviral DNA was assessed within naive (sorted using the markers above) and bulk memory (CD95^+^) CD4^+^ T cells from lymph nodes. Due to low cell recovery, our analysis in memory cells was limited to four macaques. In those animals, levels ranged from 23 to 240 intact viral genomes/million memory CD4^+^ T cells (Fig. 4d). Intact provirus was detected in naive CD4^+^ T cells from all six animals, albeit at lower levels, with a median value of 15 intact genomes/million naive CD4^+^ T cells (Fig. 4d). We used the ratio of intact provirus to total viral DNA to define the composition of the reservoir within naive CD4^+^ T cells. On average, one in every two viral DNA molecules was estimated to contain an intact genome (ratio, 1:2), suggesting that approximately 50% of total viral DNA within naive CD4^+^ cells is comprised of intact provirus (Fig. 4e). We were unable to perform a similar analysis within memory CD4^+^ T cells, as total DNA was quantified within each memory subset and not within bulk memory cells, as was done for IPDA. However, a recent study of SIV-infected adult macaques, at an age when memory cells are abundant, suggests that about 60% of lymph node-derived viral DNA is composed of intact genomes after 1 year of ART (49), a duration similar to that used in this study. Thus, although the frequency of naive CD4^+^ T cells harboring viral DNA is lower than the frequency of memory CD4^+^ T cells, the proportion of DNA that represents intact genomes is likely comparable to proportions within memory CD4^+^ T cells. Coupled with their higher abundance in the CD4^+^ T cell pool, naive cells make a substantial contribution to the persistent reservoir in SHIV.C.CH505-infected ART-treated infant macaques.

## DISCUSSION

ART-based strategies are insufficient to cure infection in pediatric cases, and because of this, novel remission strategies will be needed to impact the global prevalence of HIV-1 infection in children. It is known that pediatric immunity and HIV-1 infection are distinct from those in adults, yet preclinical studies in the pediatric setting remain limited (50–53). To address this gap, we assessed cellular and anatomical sites of viral persistence in orally SHIV.C.CH505-infected ART-treated infant rhesus macaques as a model of postnatal pediatric HIV-1 infection. We demonstrate that the dynamics of infection in this SHIV infant macaque model mirror those seen in HIV-1-infected children, including sustained high-level viremia and modest perturbation of peripheral CD4^+^ T cell frequency (37, 38, 54). Importantly, the ART regimen was effective at achieving durable suppression of plasma viral loads, making this model suitable for investigating sites of viral latency.

SHIV.C.CH505 persistence in infant macaques across distant anatomical sites was measured using several complementary detection assays. We assessed viral decay with ART by longitudinally quantifying cell-associated SHIV.C.CH505 DNA in blood and lymph node CD4^+^ T cells. Viral DNA tended to decline after ART initiation but was detectable in all but one animal even after a year on the regimen. When measuring levels of replication-competent virus in blood and spleen, we found that the spleen was more reflective of pre-ART plasma viral loads, as the two infant macaques with virus levels below the limit of detection (LOD) in the spleen QVOA were the same macaques that were refractory to challenges and had dampened plasma viral burden pre-ART. This trend is in line with previous studies implicating lymphoid organs as an early site of infection and a major source of plasma viremia in the absence of ART (55, 56). SHIV.C.CH505 persistence was also evaluated in myeloid cells isolated from the brain. Viral DNA was detected at low levels in brain CD11b^+^ cells from two infant macaques, in line with previous reports of relatively low levels of infected cells in the brains of SIV/SHIV-infected infant macaques (57). Infrequent detection of viral DNA in the brain across animals may also be reflective of a limited capacity for neuroinvasion or restricted infection of myeloid cells by SHIV.C.CH505. It will be important to explore these possibilities further in studies designed to investigate the central nervous system as a persistent reservoir in the pediatric setting (58). Nevertheless, our quantification of viral DNA at distant anatomical sites has provided insight into viral dissemination and persistence in tissues in the setting of postpartum transmission.

Even under suppressive ART, a small number of HIV/SIV-infected cells can remain transcriptionally active, capable of producing viral RNA, proteins, and virions (59–61). We identified significant transcriptional activity in the rectal compartments of ART-treated infant rhesus macaques, where ratios of cell-associated SHIV.C.CH505 RNA/DNA were roughly 1 log higher than in blood and lymph nodes, despite all sites having similar levels of viral DNA after 48 weeks of ART. This conflicts with previous reports of relatively lower viral transcription in rectal tissue of HIV-1-infected adults on ART (17, 62), which might be explained by differences in age or other aspects of study design (transmission route, virus, or type and duration of ART regimen). We also noted that viral RNA levels in the rectum remained relatively unchanged from 8 weeks to 48 weeks on ART compared to the trend for decline seen in blood during the same time frame. One study of SIV-infected adult macaques revealed only 2-fold reductions in viral RNA-positive cells within the colon and small bowel after 20 weeks of ART, compared to 2-log reductions seen in the lymph node (20), which was hypothesized to result from suboptimal drug penetration in gut tissues. While we did not address ART levels in this study, our laboratory has previously shown that the colon contains lower levels of 9-[2-(phosphonomethoxy)propyl]adenine (PMPA; cleaved from the prodrug TDF, which was used in this study) than are found in lymphoid organs in SIV-infected ART-treated infant macaques (34). However, the opposite pattern was seen for emtricitabine and dolutegravir, and it should be noted that all of these drugs target steps in the viral life cycle that precede transcription of the integrated provirus. Other mechanisms outside ART penetrance may promote elevated persistent viral RNA production in the gut of SHIV.C.CH505-infected infant macaques, such as enrichment of target cells (29, 63), immune activation, or a yet-uncharacterized site-specific extracellular milieu that affects basal transcription rates. Addressing the role of these processes in SIV/SHIV-infected infant macaques and expanding our analysis to other regions of the gut is a focus of ongoing investigation.

All memory subsets of CD4^+^ T cells have been shown to harbor HIV/SIV DNA, but levels are typically enriched within central, transitional, and effector memory cells or, in the case of lymphoid tissue, in follicular helper cells (7–9). Analysis of proviral composition under ART started during chronic HIV-1 infection has revealed similar levels of intact provirus and degrees of inducibility across resting memory CD4^+^ T cell subsets (64). In addition, a growing body of evidence also suggests a significant role in HIV-1 persistence by naive CD4^+^ T cells (13–15), a cell type that comprises the majority of the CD4^+^ T cell pool in infancy. Studies of reservoir burden across memory subsets in HIV-infected children are limited (65) but are necessary to inform whether cure strategies directed against specific cell subsets will be relevant in the pediatric setting. Here, we applied two methods to assess cellular reservoirs in SHIV.C.CH505-infected infant macaques. By quantifying levels of total cell-associated DNA in sorted naive and memory CD4^+^ T cells, we found that naive cells are infected at a lower frequency than effector memory CD4^+^ T cells in blood and T follicular helper cells in lymph nodes. However, because of their high relative abundance in infant macaques, naive CD4^+^ T cells are a major contributor to the total reservoir, comprising nearly 50% of the burden. Furthermore, by comparing measurements of total viral DNA to intact provirus in naive CD4^+^ T cells, we estimate a 1:2 ratio of intact provirus to total viral DNA. These findings are in accordance with our previous work with SIV-infected infant macaques, in which we demonstrated replication-competent virus in naive CD4^+^ T cells isolated from lymph nodes of ART-suppressed infant macaques (34). Naïve CD4^+^ T cells may represent a particularly tenacious reservoir, as they replenish to normal levels after ART initiation in children (66), have a long life span, and can differentiate into memory cells capable of expansion. While our findings for macaques will need to be confirmed for HIV-1-infected ART-suppressed children, we propose that naive CD4^+^ T cells may be crucial to HIV-1 persistence in the pediatric setting.

Limitations of our study included a small sample size of animals sacrificed on ART for tissue sampling, making it difficult to identify significant associations with viral burden and to consistently detect significant differences in the longitudinal analyses. In addition, our analysis of T cell subsets and measurements by QVOA were limited to blood and lymphoid organs due to limiting cell numbers in some tissues. Thus, we cannot draw conclusions about relative levels of replication-competent virus or cellular sources of reservoir at other tissue sites described in this study (i.e., gastrointestinal tract and brain). While our method of oral inoculations was efficient at infecting animals to be used for preclinical cure research, we recognize that this method may not capture all the complexities of breastfeeding transmission (inoculation dose, exposure frequency, immune components of breast milk, etc.). Nevertheless, this work has provided detailed insight into whole-body reservoir composition using multiple virologic assays and paves the way for more targeted studies in this tractable model.

In conclusion, we have used a rhesus macaque model to evaluate key sites of virus persistence after long-term ART in the setting of postnatal infection. We describe the rectal compartment as a transcriptionally active reservoir, suggesting that it may contribute to chronic inflammation or be an early site of virus reactivation following discontinuation of ART. This work also provides evidence to support an important role for naive CD4^+^ T cells in latency, an abundant cell type in the pediatric immune system. These findings have been obtained in a preclinical animal model relevant to contemporary HIV-1 infections in children and will serve as a basis for future studies testing both humoral and cell-mediated curative approaches targeting the HIV-1 envelope.

## MATERIALS AND METHODS

### Animals and infection.

Sixteen infant Indian rhesus macaques (Macaca mulatta), with the exclusion of Mamu-B*08^+^ and Mamu-B*17^+^ animals, were enrolled in this study. The animals were born at the Yerkes National Primate Research Center (YNPRC) to dams housed in indoor/outdoor group housing. The infants were removed from the dams when they were approximately 2 weeks old and transferred to a nursery, where they were housed in social groups for the duration of the study. The infants were fed in accordance with the YNPRC standard operating procedures (SOPs) for nonhuman primate (NHP) feeding. After being removed from the dam, infants were fed center-approved milk replacer (Similac Advance, OptiGro infant formula with iron, and/or Similac Soy Isomil OptiGro infant formula with iron; Abbott Nutrition, Columbus, OH) until 14 weeks of age. Infants are provided softened standard primate jumbo chow biscuits (jumbo monkey diet 5037; Purina Mills, St. Louis, MO) and a portion of fruit starting around 4 weeks of age. As animals aged, additional enrichment of various fresh produce was provided daily.

All animals were infected with SHIV.C.CH505.375H.dCT (also referred to as SHIV.C.CH505). This challenge stock was grown in activated primary rhesus CD4 T cells, as described previously (35). At 4 weeks of age, the first set of animals (*n *= 4) was challenged with 1.0 ml of a 1:4 dilution of challenge stock in RPMI 1640 (equivalent to 45 ng p27 antigen, 1.6 × 10^8^ viral RNA molecules, and 8.0 × 10^7^ virions) by oral administration in two consecutive doses spaced 24 h apart. If plasma viremia was not detected by 1 week postinoculation (>60 copies/ml), the dose was escalated for rechallenge of previously inoculated animals and for initial challenge of new animals (Table 1). All animals were treated in accordance with Emory University and YNPRC Institutional Animal Care and Use Committee regulations.

### Antiretroviral therapy.

All animals proceeded to treatment with a potent three-drug ART regimen at 8 weeks postinfection (see Fig. 1a). The formulation contained two reverse transcriptase inhibitors, 5.1 mg/kg of body weight tenofovir disoproxil fumarate (TDF) and 40 mg/kg emtricitabine (FTC), plus 2.5 mg/kg of the integrase inhibitor dolutegravir (DTG). The ART cocktail was administered subcutaneously once daily at 1 ml/kg. Animals used for reservoir measurements were maintained on daily ART for a minimum of 50 weeks prior to euthanasia (*n *= 6).

### Sample collection and processing.

Blood samples were collected regularly and used for complete blood counts, routine serum chemistries, and immunostaining. Peripheral blood mononuclear cells (PBMC) were isolated by density gradient centrifugation. Axillary or inguinal lymph nodes were collected throughout the study and mechanically disrupted over a 70-μm cell strainer to prepare a single-cell suspension. Rectal biopsy specimens were also collected, enzymatically digested with collagenase and DNase I for 2 h at 37°C, and then passed through a 70-μm cell strainer. Spleen, brain, and more extensive lymph node collections were done postmortem. Spleen was processed similarly to lymph node, with the addition of treatment with ammonium chloride potassium (ACK) to clear red blood cells. Brain mononuclear cells were isolated as previously described (67). In brief, brain tissue was digested in trypsin and DNase I for 30 min at 37°C, serially passed through 180-μm and 100-μm cell strainers, and then enriched for mononuclear cells by Percoll gradient experiments. All cell suspensions were washed and immediately used for downstream assays or cryopreserved in 10% dimethyl sulfoxide-fetal bovine serum (DMSO-FBS) until use.

### Immunophenotyping and cell sorting by flow cytometry.

Staining on whole blood was performed with the following monoclonal antibodies: CD3 allophycocyanin (APC)-Cy7 (SP34-2; BD Biosciences), CD8 BV711 (RPA-T8; BioLegend), and CD4 BV650 (OKT4; BioLegend). Viability dye was also included to exclude dead cells from analysis (Live/Dead Aqua; ThermoFisher). A minimum of 100,000 events were acquired on an LSR II or FACSymphony flow cytometer (BD Biosciences) driven by the FACSDiva software package. Analysis was performed using FlowJo v10 (TreeStar). T cells were gated as live CD3^+^ cells.

For cell sorting, peripheral and lymph node CD4^+^ T cells were first enriched by negative selection with the use of magnetic beads and column purification (nonhuman primate CD4^+^ T cell isolation kit; Miltenyi). Enriched CD4^+^ T cells were then stained with viability dye (Live/Dead Aqua) and previously determined volumes of the following fluorescently conjugated monoclonal antibodies: CD3 AF700 (SP34-2; BD Biosciences), CD4 BV650 (OKT4; BioLegend), CD8 APC-Cy7 (SK1; BD Biosciences), CD45RA APC (5H9; BD Biosciences), CCR7 phycoerythrin (PE)-Cy7 (3D12; BD Biosciences), CD28 ECD (CD28.2; Beckman Coulter), and CD95 PE-Cy5 (DX2; BD Biosciences). Populations for sorting were defined as follows: naive cells, CD45RA^+^ CCR7^+^ CD95^−^; central memory T (TCM)/transitional memory T (TM) cells, CD95^+^ CCR7^+^; and effector memory T (TEM) cells, CD95^+^ CCR7^−^. Lymph node cell suspensions were stained with the same antibodies as those above plus CXCR5 PE (MU5UBEE; eBioscience) and PD-1 BV421 (EH12.2H7; BioLegend) to define T follicular helper (Tfh) cells as CXCR5^hi^ PD-1^hi^. Bulk memory CD4^+^ T cells were defined as CD95^+^ after exclusion of CD45RA^+^ CCR7^+^ cells. Sorting was performed on a FACSAria LSR II (BD Biosciences) equipped with FACSDiva software.

### Plasma SHIV RNA and cell-associated DNA/RNA quantification.

Plasma viral quantification was performed as in previous studies (68, 69). Quantification of cell-associated viral DNA was done on enriched CD4^+^ T and CD11b^+^ cells with published primer/probe sets (70). Frozen CD4^+^ T cell pellets (∼100,000 cells) collected longitudinally from blood, lymph node, and rectal biopsy specimens were lysed in proteinase K and used directly in the PCR. Brain CD11b^+^ cells (600,000 to 2 million) isolated by magnetic bead purification (human CD11b microbeads; Miltenyi) and CD4^+^ T cell subsets (40,000 to 1 million cells) sorted by flow cytometry were lysed in RLT Plus containing β-mercaptoethanol (Qiagen) and column purified for DNA per the manufacturer’s instructions (All Prep DNA/RNA minikit; Qiagen). Lysates or column-purified DNA samples were then quantified for levels of SHIV gag DNA relative to cell equivalents as determined by the number of monkey albumin gene copies (71). For cell-associated viral RNA, all cell pellets (300,000 to 2 million cells) were lysed in RLT Plus containing β-mercaptoethanol, purified for RNA, and reverse transcribed (high-capacity cDNA reverse transcription kit; ThermoFisher Scientific). SHIV *gag* RNA levels were quantified and normalized to host CD4 RNA copy numbers as described elsewhere (70, 72).

All host and viral targets were detected by TaqMan assay on an ABI 7500 system in duplicate. PCR conditions have been optimized to detect a minimum of 3 copies of viral DNA or cDNA per reaction, and so the limit of detection (LOD) for each sample was calculated to be 3 SHIV *gag* copies/number of host cell equivalents or host cell RNA copies detected in the same reaction. Samples below the LOD are indicated by an open symbol on data plots.

### Quantitative viral outgrowth assay (QVOA).

Replication-competent SHIV.C.CH505 reservoirs were measured using a previously described limiting-dilution culture assay (72). In brief, CD4^+^ T cells sorted from the blood or spleen were cocultured with CEMx174 cells in dilutions ranging from 2 × 10^6^ cells per well to 4 × 10^4^ cells per well. The ratio of CEMx174 cells added to CD4 T cell cultures was 4:1 for the 2 highest dilutions, but a constant number of 1 × 10^6^ CEMx174 cells was added to all other wells. Cultures were maintained in RPMI 1640-10% FBS containing 100 U/ml interleukin 2 (IL-2; Sigma) and split every 7 days for 21 days. SHIV RNA was isolated from the culture supernatant and DNase treated. One-step real-time reverse transcription-quantitative PCR (RT-qPCR) targeting SIV *gag* was performed using an ABI 7500 real-time PCR system (Applied Biosystems) and the TaqMan fast virus 1-step master mix (ThermoFisher Scientific) using previously published primers and probes. The frequencies of infected cells were determined by the maximum-likelihood method (73) and are expressed as infectious units per million (IUPM) CD4^+^ T cells.

### IPDA.

The intact proviral DNA assay (IPDA) was used to measure the frequency of intact SHIV proviruses as described previously for HIV (47) and SIV (48) on naive and bulk memory CD4^+^ T cells isolated by fluorescence-activated cell sorting (FACS), as described above, from lymph nodes collected postmortem. Like the previously described method for measuring intact SIV genomes, the assay for measuring intact SHIV genomes consists of three multiplex droplet digital PCR (ddPCR) reactions performed in parallel: the SHIV IPDA, an assay for unintegrated 2-long-terminal-repeat (2-LTR) circles, and the copy reference/shearing assay (RPP30). The SHIV IPDA employs a duplex primer/probe mix, which specifically identifies intact proviruses based on amplicons located in two informative positions of the genome as well as two unlabeled competition probes which exclude defective proviruses that are hypermutated at positions previously identified as frequent sites of hypermutation by full-genome sequencing (48). Unintegrated 2-LTR circles were quantified using primers and probes described in the work of Policicchio et al. (74), duplexed with the IPDA *env* amplicon. The copy reference/shearing assay utilizes two amplicons in the rhesus macaque RPP30 gene to quantify cell equivalents and DNA shearing. After subtraction of unintegrated 2-LTR circles with intact *env* and correcting for DNA shearing, data were reported as the number of intact genomes per million CD4^+^ T cells. The proportion of intact genomes within naive CD4^+^ T cells was assessed by calculating the ratio of intact provirus to total viral DNA within each sample.

### Statistical analyses.

All data graphs were generated with Prism v7 or v8 (GraphPad). One-way analysis of variance (ANOVA) was used to evaluate the impact of challenge dose, sex, and Mamu-A*01 allele status on the viral set point prior to ART initiation (8 wpi). To analyze changes in cell-associated SHIV.C.CH505 DNA and RNA levels within anatomical sites, we used the nonparametric paired Wilcoxon signed-rank test. Differences in RNA to DNA log ratios between sites was assessed by the Quade test with a *post hoc* Wilcoxon test. Friedman’s test with Dunn’s correction was applied for comparisons between CD4^+^ T cell subsets in DNA levels. For analysis of intact and defective provirus levels in naive and memory CD4^+^ T cells, the Mann-Whitney test was used. A *P *of *≤*0.05 was considered statistically significant.
